# A molecular phylogeny of the genus *Drimia* (Asparagaceae: Scilloideae: Urgineeae) in India inferred from non-coding chloroplast and nuclear ribosomal DNA sequences

**DOI:** 10.1038/s41598-019-43968-z

**Published:** 2019-05-17

**Authors:** Partha S. Saha, Sumita Jha

**Affiliations:** Center of Advanced study, Department of Botany, University of Calcutta, 35, Ballygunge Circular Road, Kolkata, 700 019 West Bengal India

**Keywords:** Plant genetics, Plant molecular biology

## Abstract

The evolutionary history of the medicinally important bulbous geophyte *Drimia* (subfamily: Scilloideae) has long been considered as a matter of debate in the monocot systematics. In India the genus is represented by a species complex, however, the taxonomic delimitation among them is ill-defined till date. In the present study, a comprehensive phylogenetic relationship among Indian species of this genus has been inferred for the first time based on chloroplast DNA *trnL* intron, *rps16-trnK* intergenic spacer, *atpB-rbcL* intergenic spacer and ribosomal DNA ITS1-5.8S-ITS2 sequences, leaf morphology, anatomy, stomatal characteristics and pollen exine ornamentations. The present findings revealed the monophyletic origin of the Indian members of *Drimia* and grouped them into two possible lineages (clade- I and II). The phylogenetic tree based on cpDNA concatenated sequences further resolved the clade-I into two distinct subclades (I and II) and clarified the intraspecies relationship among the studied members. The present study suggested a strong relationship between the molecular phylogeny and the morphological characteristics of the species studied. A possible trend of evolution of two important traits: ‘type of palisade cells’ in leaf and ‘pollen exine patterns’ among the members of *Drimia* in India was also suggested.

## Introduction

The genus *Drimia* Jacq. (Asparagaceae, subfamily Scilloideae, tribe Urgineeae sensu APG III^1^) (alternatively Hyacinthaceae subfamily Urgineoideae sensu APG II^2^) comprises approximately 110 bulbous geophytic species^3,4^ distributed in Africa, Madagascar, the Mediterranean basin and Asia^5^. The majority of the species (~93) are native to Africa. Currently, a total of eight species of the genus *Drimia* have been recognized in India viz. *D*. *coromandeliana* (Roxb.) Lekhak & P. B. Yadav, *D*. *govindappae* (Boraiah & Fatima) Lekhak & P. B. Yadav, *D*. *indica* (Roxb.) Jessop, *D*. *nagarjunae* (Hemadri and Swahari) Anand Kumar, *D*. *polyantha* (Blatt. & McCann) Stearn, *D*. *raogibikei* (Hemadri) Hemadri, *D*. *razii* Ansari and *D*. *wightii* Lakshmin^6^. Among them, seven species are endemic to the subcontinent^6,7^. Squill (European squill, *D*. *maritima*) is one of the most ancient medicinal plants. Since Stoll *et al*.^8^ isolated and crystallized scillaren A, a large number of bufadienolides have been reported from the bulbs of squill^9^. Bufadienolides (a class of cardiac glycosides) are the C-24 steroids with an α-pyrone group at position 17β^9–11^. The principle bufadienolides, i.e. scillaren A and proscillaridin A, isolated from Indian squill, *D*. *indica*^10,12–15^ are the same as those of the European squill, *D*. *maritima*^8,10,16,17^. Different species of *Drimia* show remarkable morphological similarities resulting in taxonomic misinterpretations^5,18–21^.

Several taxonomic revisions of Indian members of *Drimia* have been published^18,22–29^, relying solely on morphological characters for species delimitation^6^. Lekhak *et al*.^7^ and Yadav *et al*.^6^ inferred that, morphological characterization alone may not be sufficient to delimit interspecies relationship in this genus. To address this problem, a few studies have been conducted so far based on cytotaxonomy, karyotype, palynology, interspecific hybridization, nuclear DNA content, RAPD and SRAP markers, ITS and *mat*K sequence data^6,7,30–33^. However, the phylogenetic relationships among the Indian members of *Drimia* still remain unclear.

Molecular phylogenetic studies have conventionally relied on comparison of homologous nucleotide sequences to establish a degree of similarity between closely related species. The use of nuclear and/or organellar non-coding sequences has greatly assisted our understanding of relationships and circumscriptions at all levels of the taxonomic hierarchy in plant phylogenetic studies^34–38^. Analysis of plastid DNA sequences has proven to be very useful in the phylogenetic study of Hyacinthaceae^5^. The potential use of leaf anatomical characteristics in the species level phylogeny has also been well documented in different monocot plant groups, particularly in Hyacinthaceae^39–41^. As far as we are aware, no comparative study has been carried out on leaf morpho-anatomical features of Indian species of *Drimia*. The stomatal traits of the monocot leaves have been considered as important taxonomic markers in different levels of systematic hierarchy^37,42^. Similarly, pollen grain characteristic, especially the exine micromorphology has also been reported to be very useful in defining the evolutionary trends in many plant families^43–46^.

The aim of the present study is to infer the phylogenetic relationships within the Indian species of *Drimia* based on cpDNA *trnL* intron, *rps16-trnK* intergenic spacer, *atpB-rbcL* intergenic spacer and rDNA ITS1-5.8S-ITS2 sequences, along with leaf morpho-anatomical, stomatal and pollen exine micromorphological characteristics.

## Results

Out of the eight Indian species^6^, the present study deals with 12 accessions representing seven species of *Drimia* (Table 1). To investigate the phylogenetic relationships cpDNA non-coding (*trnL* intron, *rps16-trnK* intergenic spacer and *atpB-rbcL* intergenic spacer) and nuclear rDNA ITS1-5.8S-ITS2 sequences of the collected species of this genus were analysed along with the characterization of leaf morpho-anatomical and pollen exine morphological features.

**Table 1:** List of collected Indian species of *Drimia* with their site of collection, somatic chromosome number^7,32,33,51^ and NCBI GenBank accession numbers.

Sl. No.	Species with author citation	Population	Voucher No.	Site of collection	Somatic chromosome no. (2n)^7,32,33,51^	GenBank Accession ID			
						cpDNA *trnL* intron	cpDNA *rps16-trnK* intergenic spacer	cpDNA *atpB-rbcL* intergenic spacer	rDNA ITS1-5.8S-ITS2
1	*D*. *indica* (Roxb.) Jessop	Population I	SUK-5282	Sanaghagara, Keonjhar District, Odisha	20	MK047596	MK069653	MK069662	MK087144
		Population II	SUK-031	Sindhudurg District, Maharashtra	20	MK047597	MK069654	MK069663	MK087644
		Population III	DIR	Jodhpur, Rajasthan	20	MK047598	MK069655	MK069664	MK087672
		Population IV	DIW3	Kolhapur, Maharashtra	30	MK047599	MK069656	MK069665	MK088176
2	*D*. *coromandeliana* (Roxb.) Lekhak & P. B. Yadav	—	SUK-5284	Kagal, Kolhapur, Maharashtra	40	MK163337	MK163338	MK163339	MK129258
3	*D*. *polyantha* (Blatt. & McCann) Stearn	Population I	SUK-5283	Kolhapur, Maharashtra	20	MK047600	MK069657	MK069666	MK088175
		Population II	DPW2	Talewadi, Maharashtra	20	MK047601	MK069658	MK069667	MK087862
4	*D*. *razii* Ansari	—	SUK-5285	Diveghat, Pune, Maharashtra	20	MK047602	MK069659	MK069668	MK088055
5	*D*. *wightii* Lakshmin	Population I	SUK-5292	Nesari, Kolhapur, Maharashtra	20	MK047603	MK069660	MK069669	MK088056
		Population II	DW2	Halkarni, Maharashtra	20	MK047604	MK069661	MK069670	MK088067
6	*D*. *nagarjunae* (Hemadri and Swahari) Anand Kumar	—	MML-445	Chinnar wildlife sanctuary, Idukki district, Kerala	20	MK113830	MK113832	MK113834	MK129257
7	*D*. *govindappae* (Boraiah & Fatima) Lekhak & P. B. Yadav	—	SUK-032	Bangalore, Karnataka	20	MK113829	MK113831	MK113833	MK129262

### Phylogeny of *Drimia* (subfamily Scilloideae) inferred from cpDNA *trnL* intron sequences

**Figure 1:**
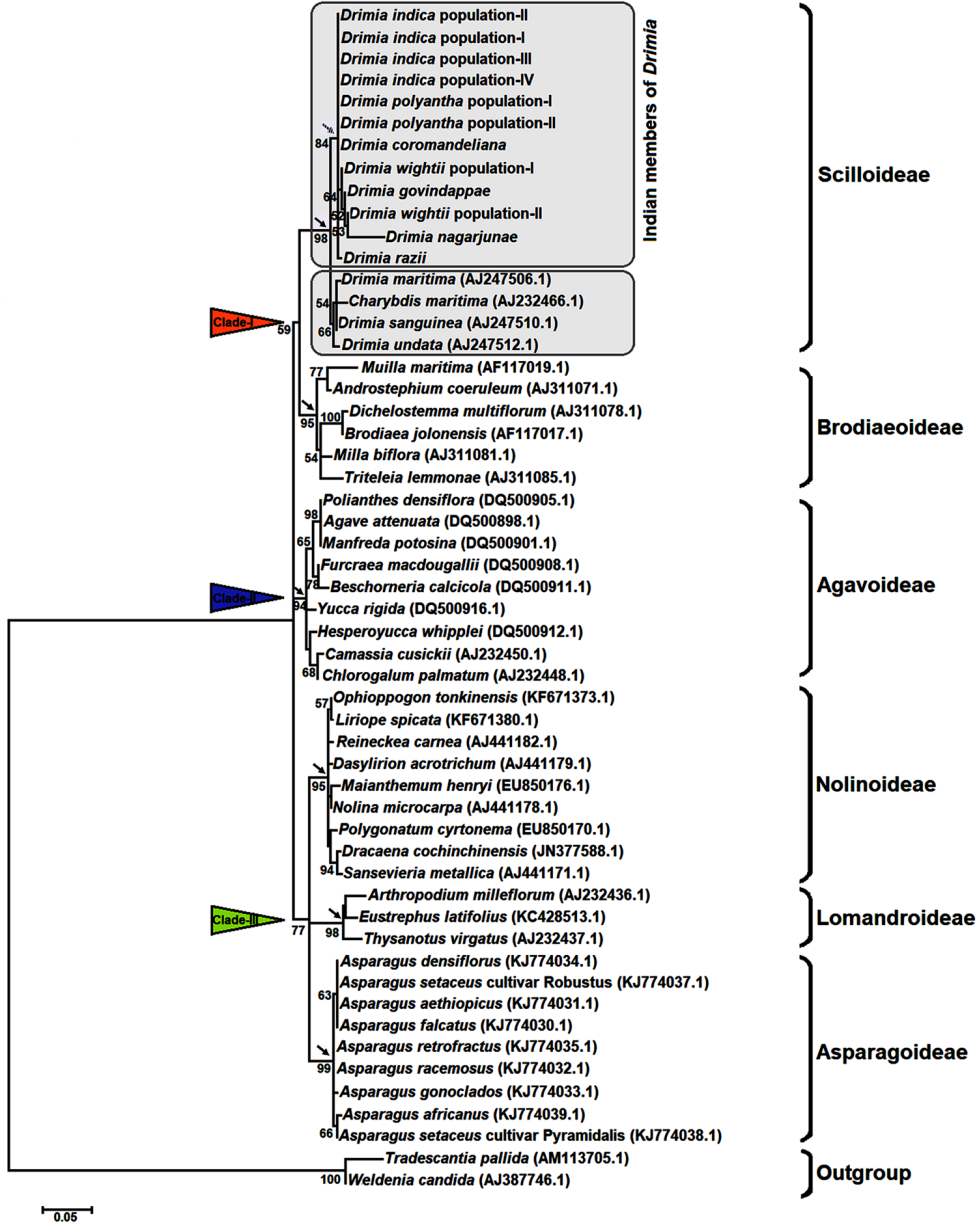
Maximum likelihood phylogeny of the genus *Drimia* (subfamily Scilloideae) based on cpDNA *trnL* intron sequences. Numbers beneath nodes are Bootstrap support (BS) indices. Black arrows indicate formation of six subclades. Dotted arrow indicates the origin of all studied Indian members of *Drimia* from a common node.

The maximum likelihood (ML) phylogenetic tree comprising a total of 54 taxa was rooted with two closely related outgroup taxa^47^ i.e. *Tradescantia pallida* and *Weldenia candida* (Fig. 1). All ingroup members representing six different subfamilies of the family Asparagaceae fell into three major clades (I, II and III). The clade-I was subdivided into two subclades representing Scilloideae and Brodiaeoideae with bootstrap values (BS) 98% and 95% respectively. The clade-II consisted of only Agavoideae (BS 94%), while clade-III was subdivided into three subclades representing Nolinoideae (BS 95%), Lomandroideae (BS 98%) and Asparagoideae (BS 99%). In subfamily Scilloideae of clade-I, all Indian members of *Drimia*, viz. four populations of *D*. *indica*, two populations each of *D*. *polyantha* and *D*. *wightii*, and the single populations of *D*. *coromandeliana*, *D. govindappae, D*. *nagarjunae* and *D*. *razii* grouped together and originated from a single node (BS 84%), supporting the monophyly of the Indian members. Within the clade, *D*. *wightii*, *D*. *govindappae* and *D*. *nagarjunae* grouped with weak support (BS 64%) (Fig. 1). The three non-Indian species (*D*. *maritima* and *D*. *undata* from Europe and *D*. *sanguinea* from southern Africa) were weakly supported (BS 54%) as sister to the Indian species (Fig. 1).

### Phylogenetic relationships among the Indian species of *Drimia* based on concatenated sequences of cpDNA *rps16-trnK* intergenic spacer, *atpB-rbcL* intergenic spacer and *trnL* intron

**Figure 2:**
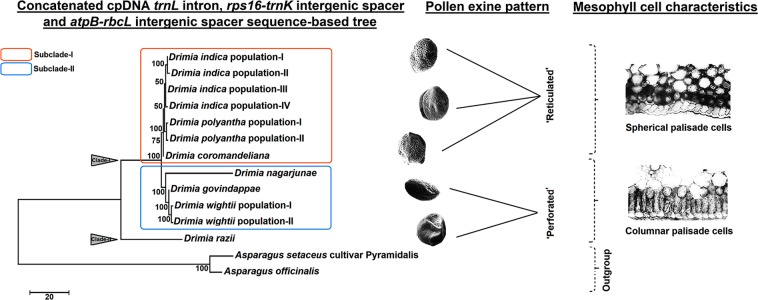
Maximum parsimony phylogeny among the Indian species of *Drimia* based on concatenated sequences of cpDNA *trnL* intron, *rps16-trnK* intergenic spacer and *atpB-rbcL* intergenic spacer. Numbers beneath nodes are Bootstrap support (BS) indices. Two selected morphological characters of taxonomic importance (pollen exine pattern and mesophyll cell characteristics) have been mapped on the tree.

In order to clarify the interspecies relationships among the Indian members of the genus *Drimia*, a phylogenetic tree based on combined sequences of cpDNA *rps16-trnK* intergenic spacer, *atpB-rbcL* intergenic spacer and *trnL* intron was reconstructed. Both MP and ML methods yielded identical topologies (only MP tree is shown in Fig. 2). The Indian species of *Drimia* were split into two distinct clades (Fig. 2). The clade-I was subdivided into two subclades (I- II), where subclade-I included *D*. *coromandeliana*, *D*. *indica* and *D*. *polyantha*, while the subclade-II included *D*. *govindappae*, *D*. *nagarjunae* and *D*. *wightii*. This topology was similar to the cpDNA *trnL* intron-based tree (Fig. 1). Among the members of subclade-I, all four populations of *D*. *indica* clustered together, while both the populations of *D*. *polyantha* originated from a single node (BS 100%) and *D*. *coromandeliana* emerged as a sister taxon. On the other hand, the clade-II of the cpDNA concatenated sequence-based tree consisted of *D*. *razii* (Fig. 2).

### Phylogenetic relationships among the Indian species of *Drimia* based on rDNA ITS1-5.8S-ITS2 sequence

Phylogenetic relationships among the Indian species of *Drimia* were also assessed based on rDNA ITS1-5.8S-ITS2 sequences. The ML tree (Supplementary Fig. S1) resolved two distinct clades (I- II). The clade-I (BS 100%) comprising *D*. *coromandeliana*, *D*. *indica* and *D*. *polyantha* (Supplementary Fig. S1) was similar to that of the subclade-I of the cpDNA concatenated sequence-based tree (Fig. 2). The remaining species of *Drimia* i.e. *D*. *nagarjunae*, *D*. *govindappae*, *D*. *wightii* and *D*. *razii* formed the clade-II (BS 78%) on the basis of their rDNA ITS1-5.9S-ITS2 sequence complexity (Supplementary Fig. S1).

### Morpho-anatomical and stomatal characteristics of leaf

Various qualitative and quantitative morphological parameters of leaf were evaluated (Supplementary Table S1). All Indian species of *Drimia* are characterized by green or glaucous, fleshy leaves. The species vary in the number of leaves per bulb (LN) (Table 2). The greatest number of leaves per bulb (13.8 ± 0.29 to 14.2 ± 0.24) was found in the four populations of *D*. *indica*, while *D*. *razii* has the fewest (6.2 ± 0.35). Three major types based on leaf shape (LS) i.e. lanceolate-straight (0), linear (1) and lanceolate-curled (2) were observed among the studied taxa. The leaves in *D*. *indica*, *D*. *coromandeliana*, *D*. *polyantha* and *D*. *nagarjunae* are lanceolate-straight [character state = 0], linear [character state = 1] in *D*. *razii*, and lanceolate-curled in *D*. *wightii* and *D*. *govindappae* [character state = 2] (Supplementary Fig. S2a; Table 2). The longest leaves (43.9 ± 0.64 cm) were recorded in *D*. *indica* population-IV and the widest in *D*. *nagarjunae* (4.0 ± 0.02 cm) while *D*. *razii* had the shortest (21.1 ± 0.38 cm) and narrowest (0.2 ± 0.0 cm) (Table 2).

**Table 2:** Morphological and anatomical characterization of leaves of 12 Indian accessions of Drimia*.

Species	Morphological characters of leaf				Anatomical characters of leaf			
	Qualitative		Quantitative		Qualitative		Quantitative	
	LS	LN	LL (in cm)	LW (in cm)	LS_T.S_	PT	LPC (in µm)	LT_T.S_ (in µm)
*D*. *indica* pop-I	0	14.0 ± 0.51^f^	43.3 ± 0.90^f^	2.8 ± 0.02^d^	0	1	9.5 ± 0.08^a^	271.9 ± 0.18^f^
*D*. *indica* pop-II	0	14.2 ± 0.24^f^	43.8 ± 0.48^f^	2.9 ± 0.04^d^	0	1	9.5 ± 0.05^a^	272.0 ± 0.10^f^
*D*. *indica* pop-III	0	14.0 ± 0.39^f^	43.1 ± 0.64^f^	2.9 ± 0.05^d^	0	1	9.5 ± 0.06^a^	272.4 ± 0.10^f^
*D*. *indica* pop-IV	0	13.8 ± 0.29^f^	43.9 ± 0.64^f^	2.9 ± 0.04^d^	0	1	9.5 ± 0.04^a^	272.2 ± 0.15^f^
*D*. *coromandeliana*	0	10.8 ± 0.32^e^	37.7 ± 0.50^d^	2.1 ± 0.03^c^	0	1	10.0 ± 0.05^b^	244.5 ± 0.13^e^
*D*. *polyantha* pop-I	0	9.3 ± 0.21^c,d^	31.3 ± 0.46^c^	2.0 ± 0.02^c^	0	1	9.3 ± 0.03^a^	202.0 ± 0.17^d^
*D*. *polyantha* pop-II	0	9.4 ± 0.30^d^	30.8 ± 0.46^c^	1.9 ± 0.03^c^	0	1	9.5 ± 0.04^a^	202.1 ± 0.18^d^
*D*. *razii*	1	6.2 ± 0.35^a^	21.1 ± 0.38^a^	0.2 ± 0.00^a^	1	0	22.8 ± 0.18^f^	334.0 ± 0.11^g^
*D*. *wightii* pop-I	2	8.3 ± 0.21^b,c,d^	25.2 ± 0.27^b^	1.5 ± 0.03^b^	0	0	16.4 ± 0.11^c^	104.1 ± 0.10^a^
*D*. *wightii* pop-II	2	8.0 ± 0.21^b,c^	26.8 ± 0.33^b^	1.5 ± 0.02^b^	0	0	16.4 ± 0.06^c^	104.0 ± 0.10^a^
*D*. *nagarjunae*	0	8.0 ± 0.25^b,c^	40.9 ± 0.34^e^	4.0 ± 0.02^e^	0	0	21.5 ± 0.10^d^	154.2 ± 0.10^b^
*D*. *govindappae*	2	7.2 ± 0.29^a,b^	26.4 ± 0.36^b^	1.6 ± 0.04^b^	0	0	22.0 ± 0.21^e^	161.2 ± 0.15^c^

*Values (Mean ± S.E.) followed by same letter are not significantly different; according to Tukey’s B multiple range tests (P = 0.05). LS: Shape of leaf (0 = lanceolate-straight; 1 = linear; 2 = lanceolate-curved); LN: Number of leaves per bulb; LL: Leaf length; LW: Leaf width; LS_T.S_: Shape of leaf in t.s. (0 = subulate; 1 = polygonal); PT: Type of palisade cells (0 = columnar; 1 = spherical); LPC: Length of palisade cells; LT_T.S_: Thickness of leaf in t.s.

Several qualitative and quantitative anatomical characters were also studied (Supplementary Table S1). The species were found to vary in the cross-sectional shape of the leaves (LS_T.S_) (Table 2). The leaves of all species except *D*. *razii* were subulate in shape [character state = 0] whereas leaves of *D*. *razii* were polygonal in section [character state = 1] (Table 2). Anatomically, the basic leaf features of the genus *Drimia* include the presence of a thick cuticle, an epidermis of barrel or rectangular shaped cells, and a series of chlorenchymatous mesophyll tissues. The mesophyll tissues could be further categorized into two types i.e. compact, single layered palisade cells and loosely arranged irregularly shaped, multiple layered spongy cells with intercellular spaces aligned horizontally next to the inner layer of palisade cells. In addition, two types of palisade cells (PT) were observed among the studied samples, i.e., columnar [character state = 0] in *D*. *wightii*, *D*. *razii*, *D*. *govindappae*, *D*. *nagarjunae* (Supplementary Fig. S2b; Table 2) and spherical types [character state = 1] in *D*. *indica*, *D*. *coromandeliana* and *D*. *polyantha* (Supplementary Fig. S2c; Table 2). The length of palisade cells (LPC) varied significantly among the species (Table 2). The maximum LPC (22.8 ± 0.18 μm) was observed in *D*. *razii* followed by *D*. *govindappae* (22.0 ± 0.21 μm) and *D*. *nagarjunae* (21.5 ± 0.10 μm). *Drimia polyantha* and *D*. *indica* showed minimum LPC (ranging from 9.3 ± 0.03 μm to 9.5 ± 0.08 μm). The collateral vascular bundles were found at regular intervals, with adaxial phloem and relatively well-developed xylem abaxially. One to two layers of compactly arranged spherical bundle sheath cells were found around the vascular bundles of each species. Additionally, significant differences in leaf thickness in cross-section (LT_T.S_) ranging from 104.0 ± 0.10 μm to 334.0 ± 0.11 μm were observed (Table 2).

**Figure 3:**
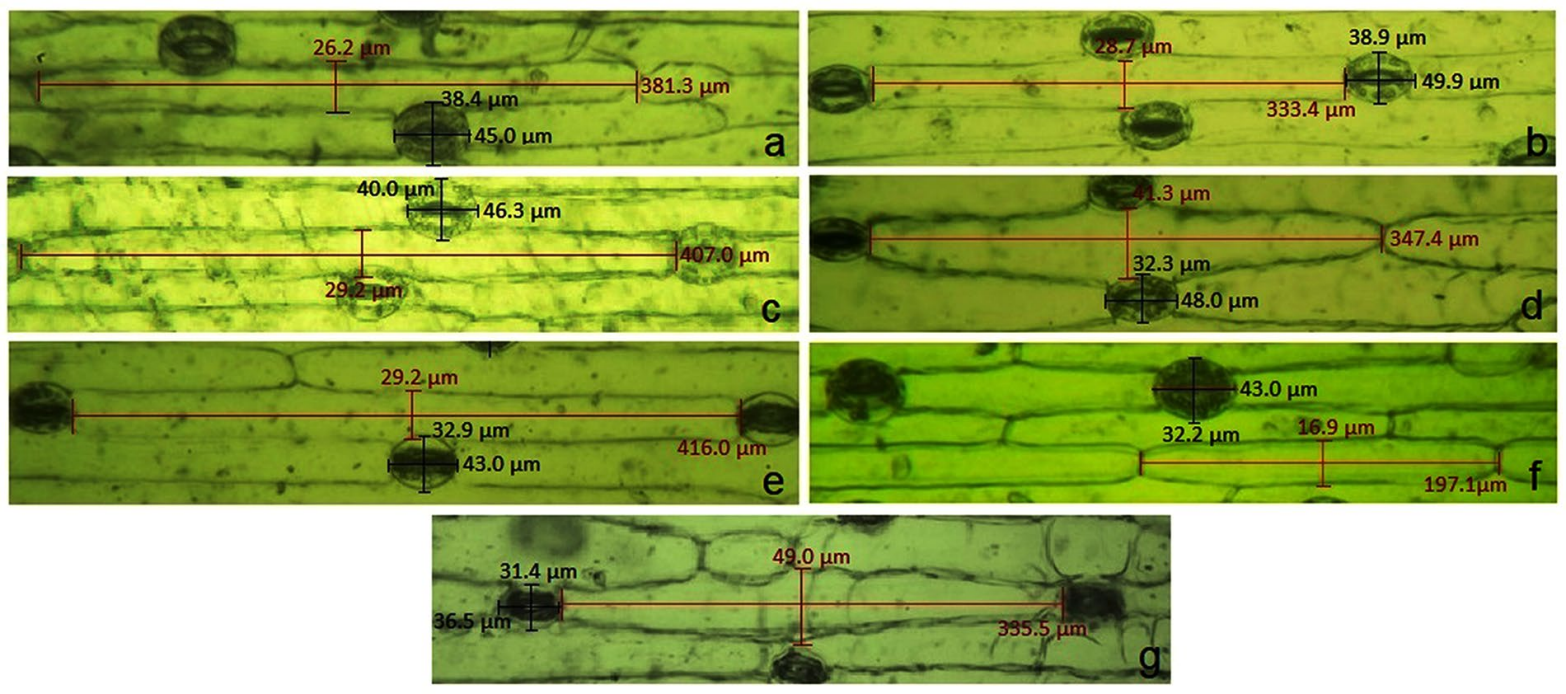
Stomatal characteristics of seven Indian species of *Drimia*. (**a**) *D*. *indica* population-I; (**b**) *D*. *coromandeliana*; (**c**) *D*. *razii;* (**d**) *D*. *polyantha* population-I; (**e**) *D*. *nagarjunae*; (**f**) *D*. *wightii* population-I; (**g**) *D*. *govindappae*.

Stomatal traits are considered as one of the important taxonomic markers in delimiting species^37,48^. The present study revealed that all taxa were characterized by anomocytic type (without subsidiary cells) of stomata (Fig. 3a–g). However, the length and breadth of stomata were found to be species-specific (Table 3, Fig. 3a–g). The stomatal index (SI) varied approximately 1.8-fold among the species (Table 3). Maximum SI was recorded in *D*. *polyantha* while *D*. *indica* and *D*. *wightii* showed minimum SI (Table 3). The species also differed in the length (EL) and width (EW) of the surrounding epidermal cells. Maximum EL was recorded in *D*. *nagarjunae* (416.0 ± 0.04 μm; Fig. 3e) and minimum EL (197.1 ± 0.08 μm) and EW (16.9 ± 0.08 μm) in population-I of *D*. *wightii* (Fig. 3f; Table 3).

**Table 3:** Stomatal characterization of leaves of 12 Indian accessions of Drimia*.

Species	Stomatal characters				
	SL (in µm)	SW (in µm)	SI	EL (in µm)	EW (in µm)
*D*. *indica* pop-I	45.0 ± 0.05^c^	38.4 ± 0.09^e^	23.4 ± 0.26^a^	381.3 ± 0.06^e^	26.2 ± 0.05^b^
*D*. *indica* pop-II	45.2 ± 0.16^c^	38.4 ± 0.06^e^	23.2 ± 0.16^a^	381.5 ± 0.09^e^	26.1 ± 0.07^b^
*D*. *indica* pop-III	45.0 ± 0.19^c^	38.6 ± 0.07^e,f^	24.0 ± 0.28^a^	381.0 ± 0.15^e^	26.1 ± 0.13^b^
*D*. *indica* pop-IV	45.0 ± 0.22^c^	38.5 ± 0.04^e,f^	23.7 ± 0.25^a^	381.0 ± 0.17^e^	26.1 ± 0.12^b^
*D*. *coromandeliana*	49.9 ± 0.05^f^	38.9 ± 0.20^f^	33.0 ± 0.33^c^	333.4 ± 0.07^b^	28.7 ± 0.11^c^
*D*. *polyantha* pop-I	48.0 ± 0.08^e^	32.3 ± 0.05^c^	41.0 ± 0.45^e^	347.4 ± 0.06^d^	41.3 ± 0.06^e^
*D*. *polyantha* pop-II	48.0 ± 0.18^e^	31.7 ± 0.08^b^	42.0 ± 0.49^e^	347.2 ± 0.39^d^	41.4 ± 0.05^e^
*D*. *razii*	46.3 ± 0.07^d^	40.0 ± 0.06^g^	30.5 ± 0.20^b^	407.0 ± 0.11^f^	29.2 ± 0.06^d^
*D*. *wightii* pop-I	43.0 ± 0.06^b^	32.2 ± 0.09^c^	24.1 ± 0.15^a^	197.1 ± 0.08^a^	16.9 ± 0.08^a^
*D*. *wightii* pop-II	43.0 ± 0.21^b^	31.3 ± 0.06^a^	24.1 ± 0.15^a^	197.2 ± 0.15^a^	16.9 ± 0.15^a^
*D*. *nagarjunae*	43.0 ± 0.06^b^	32.9 ± 0.08^d^	36.0 ± 0.30^d^	416.0 ± 0.04^g^	29.2 ± 0.07^d^
*D*. *govindappae*	36.5 ± 0.07^a^	31.4 ± 0.07^a,b^	35.2 ± 0.26^d^	335.5 ± 0. 08^c^	49.0 ± 0.07^f^

^*^Values (Mean ± S.E.) followed by same letter are not significantly different; according to Tukey’s B multiple range tests (P = 0.05). SL: Stomatal length; SW: Stomatal width; SI: Stomatal index; EL: Epidermal cell length; EW: Epidermal cell width.

### Pollen exine morphology based on scanning electron microscopic (SEM) study

**Figure 4:**
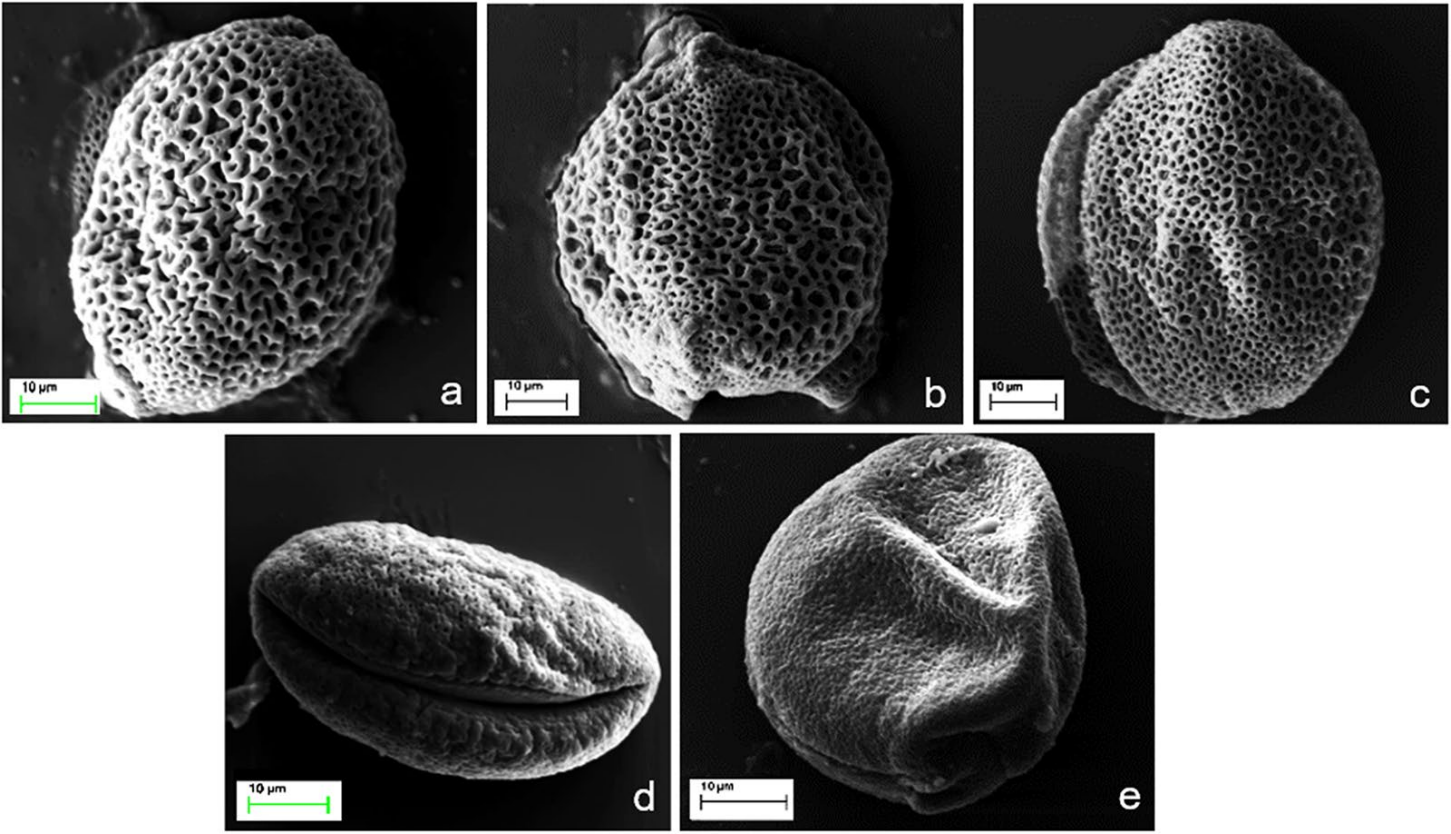
Scanning electron microscopic images showing pollen exine ornamentation patterns in five Indian species of *Drimia*. (**a**–**c**) Reticulate exine in *D*. *indica* population-I, *D*. *coromandeliana* and *D*. *polyantha* population-I; (**d**–**e**) Perforate exine in *D*. *govindappae* and *D*. *wightii* population-I.

Pollen morphological traits have been used as taxonomic markers in Hyacinthaceae^19,49^. In this study we examined the exine surface architectures of pollen grains of all the collected species of *Drimia* except *D*. *razii* and *D*. *nagarjunae* (in which flowering was not observed) under SEM. All species had monosulcate, ellipsoidal grains (Fig. 4) but two distinct types of exine ornamentation were observed. Reticulate exine [character state = 1] was observed in *D*. *indica*, *D*. *coromandeliana* and in *D*. *polyantha* (Fig. 4a–c) while pollen grains of *D*. *wightii* and *D*. *govindappae* were characterized by perforate exine [character state = 0] (Fig. 4d and 6e). An earlier report on *D*. *razii* pollen grains showed them to be of the perforate type (or fine reticulate)^19^ and this was species was therefore coded as ‘character state = 0’ and used for further analysis on ancestral state reconstruction.

### Interspecies relationship based on UPGMA phenogram analysis using combined leaf morpho-anatomical and stomatal data

**Figure 5:**
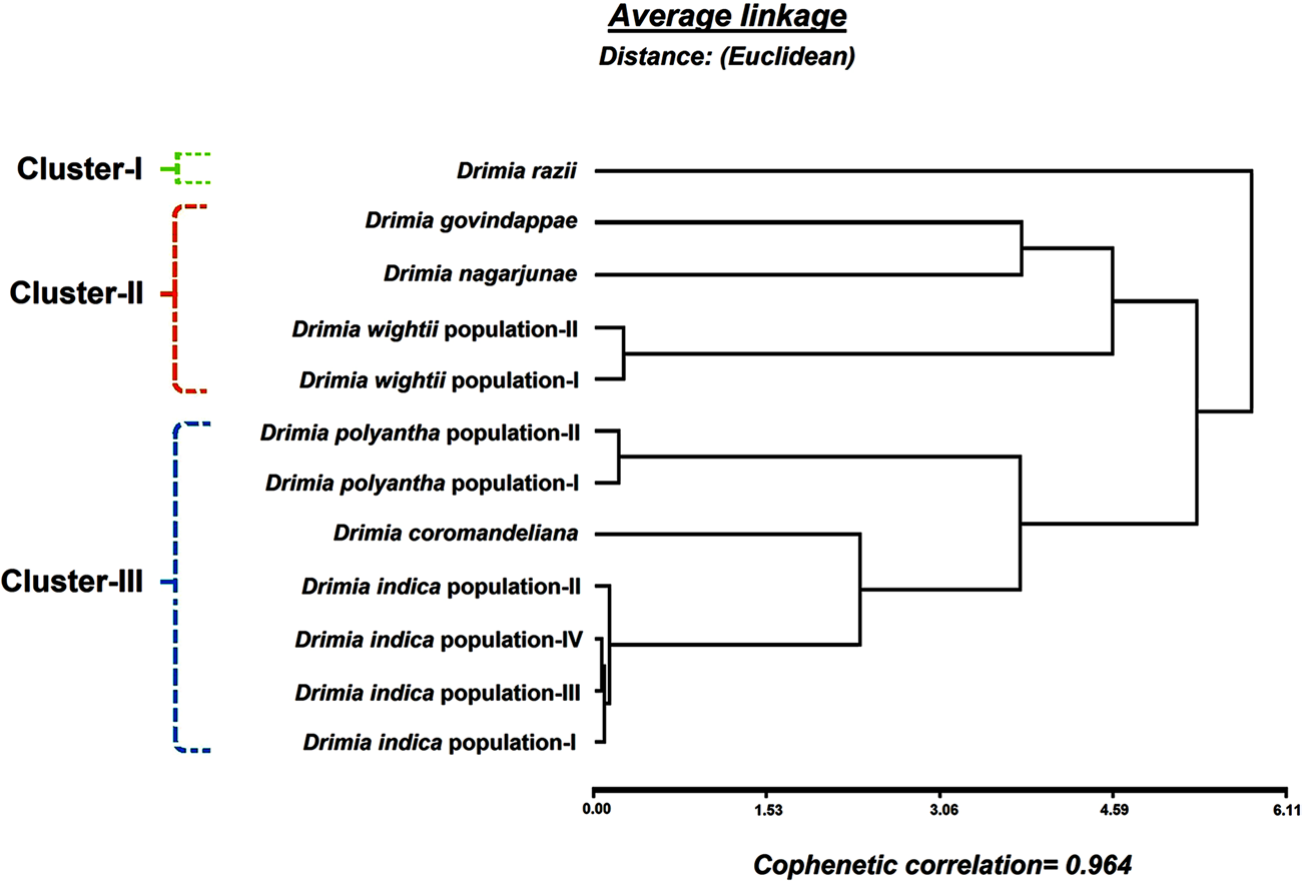
UPGMA phenogram derived from average Euclidean distance between taxa of the genus *Drimia* using combined morphological, anatomical and stomatal data.

The cophenetic correlation for the obtained UPGMA phenogram (Fig. 5) was 0.964, indicating a good fit between the cophenetic value matrix and the average Euclidean distance matrix. The observed phenogram (Fig. 5) revealed the formation of three distinct clusters (I-III). The cluster-I consisted of *D*. *razii*, while the cluster-II included *D*. *govindappae*, *D*. *nagarjunae* and two populations of *D*. *wightii*. The cluster-III was composed of two populations of *D*. *polyantha*, *D*. *coromandeliana* and four populations of *D*. *indica* (Fig. 5) which was similar to the subclade-I of the cpDNA concatenated sequence-based tree (Fig. 2).

### Ancestral state assessment for type of palisade cell (PT) and pollen exine pattern (PEP)

We reconstructed the ancestral state for the type of palisade cells (PT) of leaf and pollen exine patterns (PEP) in Indian *Drimia*. A phylogenetic tree based on combined sequences of cpDNA *trnL* intron, *rps16-trnK* intergenic spacers and *atpB-rbcL* intergenic spacers was constructed and used as a backbone for tracing ancestral character (Supplementary Figs S3 and S4). The obtained maximum parsimony (MP) tree, rooted with *D*. *razii*, revealed the distribution of character states in the terminal taxa and the evolutionary history of both leaf and pollen characters was studied. The ancestral state reconstruction clearly showed that columnar palisade cells (Supplementary Fig. S3) and perforate exine of pollen (Supplementary Fig. S4) were the ancestral characters in Indian *Drimia* species. The exine ornamentation in *D*. *nagarjunae* was not observed in the present study. The results revealed that *D*. *razii*, *D*. *wightii*, *D*. *govindappae* and *D*. *nagarjunae* retained the ancestral states for leaf palisade cell characters, while *D*. *polyantha*, *D*. *coromandelian* and *D*. *indica* shared derived spherical palisade cells (Supplementary Fig. S3) and reticulate pollen exine architecture (Supplementary Fig. S4).

## Discussion

This study demonstrated, for the first time, an explicit phylogenetic relationship among seven different Indian species of *Drimia* on the basis of cpDNA non-coding sequences (*trnL* intron, *rps16-trnK* intergenic spacer and *atpB-rbcL* intergenic spacer), rDNA ITS1-5.8S-ITS2 sequence data, leaf morpho-anatomical, stomatal data and pollen exine morphological data.

Our analysis shows that the Indian species of *Drimia* that were sampled comprise a monophyletic group derived from a common ancestor (Fig. 1), thereby supporting the theory that India may be a secondary centre of evolution for this genus^19^. A previous analysis of systematic relationship among five Indian taxa of *Drimia* based on ITS and *mat*K DNA sequence variations^30^ did not include the two important Indian taxa i.e. *D*. *govindappae* and *D*. *nagarjunae*, and may require re-evaluation. The present study included 12 accessions representing seven Indian species of *Drimia* and was made on the basis of concatenated sequences of cpDNA *trnL* intron *rps16-trnK* intergenic spacers and *atpB-rbcL* intergenic spacers (Fig. 2), and was further confirmed by analysing the rDNA ITS1-5.8S-ITS2 sequence-based tree (Supplementary Fig. S1). The phylogenetic trees rooted with two *Asparagus* species (outgroups) retrieved, *D*. *razii* as sister to the other six species, which grouped into two evolutionary lines, providing deeper insights into the interspecies relationships in the genus in India. Nath *et al*.^33^ also suggested the grouping of all the Indian species of *Drimia* into two major complexes based on karyological data.

Boraiha and Khaleel^27^ first recognized *D*. *govindappae* as distinct from *D*. *indica* and *D*. *coromandeliana*, although Dixit and Yadav^18^ reported successful hybridization between *D*. *indica* and *D*. *govindappae*. However, intermediate forms of these species have not been observed in nature so far. Based on floral morphology, Deb and Dasgupta^25^ treated *D*. *coromandeliana*, *D*. *nagarjunae* and *D*. *govindappae* as synonyms of *D*. *indica*. On the contrary, the present UPGMA analysis (Fig. 5) clearly demarcates these species on the basis of combined leaf morphological, anatomical and stomatal data. The UPGMA phenogram revealed the formation of three distinct clusters (I- III) among the studied Indian taxa of *Drimia* (Fig. 5), which is positively correlated with the results obtained from the molecular phylogenetic tree (Fig. 2).

The Indian species of *Drimia* include both day-blooming and night-blooming species with two types of life cycle patterns, viz. synanthous (leaves and flowers appeared simultaneously) and hysteranthous (leaves and flowers appear in different seasons)^6,7,50^. Yadav and Dixit^19^ observed peculiarity in the time of flower opening and closing among the closely related *Drimia* species. Differences in the timing of anthesis among the different taxa of *Drimia* also induced troubles in the hybridization experiments^51^. Peruzzi *et al*.^40^ discussed the utility of several leaf anatomical traits including different types of palisade cells in the grouping of different species of the genus *Ornithogalum* (Hyacinthaceae). The present study confirms the value of stomatal characteristics in interspecies delimitations among the genus *Drimia* in India. In our earlier work on *Asparagus*, the evolutionary significance of different stomatal as well as the surrounding epidermal cell traits in species-level phylogeny of the subgenus *Protasparagus* was clearly demonstrated^37^. Pollen exine morphology was highly congruent with molecular data (Fig. 2). Yadav and Dixit^19^ studied the pollen exine ornamentations of four Indian species and categorized them accordingly. Pehlivan and Özler^49^ characterized different taxa of *Muscari* (Hyacinthaceae) on the basis of pollen surface ornamentation.

The present phylogenetic analysis also sheds light on the evolution of two important leaf and pollen characters i.e. type of palisade cells (PT) and pollen exine patterns (PEP) of the Indian species of *Drimia* (Supplementary Figs S3 and S4). The parsimony ancestral states reconstruction using the combined sequences of cpDNA *trnL* intron *rps16-trnk* intergenic spacer and *atpB-rbcL* intergenic spacer suggested that the columnar palisade cell is ancestral in *Drimia* in India and spherical palisade cells are derived. Similarly, a possible trend of evolution from perforate to reticulate pollen exine ornamentation is also suggested in the present analysis. However, further investigations on different vegetative and floral characters of the genus *Drimia* including *D*. *nagarjunae* pollen grains and allied members of Scilloideae are needed to infer evolutionary significance of the present observation.

In conclusion, the present research work demonstrates an explicit phylogenetic relationship among seven Indian species of *Drimia* on the basis of both molecular and leaf morpho-anatomical characters for the first time. This study also highlights the possible evolution of exine ornamentation. Altogether, the present research work brings out new insights on species diversification of *Drimia* in India and provides important background information for further studies on their biogeography.

## Methods

### Taxon sampling

Out of eight Indian species recognized by Yadav *et al*.^6^, a total of 12 accessions representing seven species of *Drimia* were used in the present study (Table 1). Voucher specimens were deposited in the Herbarium of Shivaji University, Kolhapur (SUK). All the samples (bulbs) have been grown and maintained for more than eight years in the net house at the experimental garden of the Department of Botany, University of Calcutta (elev. 9 m, 22.5275° N, 88.3628° E). A representative number of individual plants for each taxon adapted in a similar environment have been used for the present phylogenetic analysis. A total of 54 accessions representing all the subfamilies of Asparagaceae (except the monogeneric subfamily Aphyllanthoideae) were analysed for cpDNA *trnL* intron sequence-based phylogeny of the genus *Drimia*. Among them, sequences of 42 accessions representing five subfamilies of Asparagaceae (Brodiaeoideae, Agavoideae, Asparagoideae, Lomandroideae and Nolinoideae) including two outgroup taxa (*Tradescantia pallida*: Accession no.: AM113705.1 and *Weldenia candida*: Accession no.: AJ387746.1) were retrieved from the NCBI public database (http://www.ncbi.nim.nih.gov) (Supplementary Table S2) following the taxonomic classification of APG III^47^.

### Genomic DNA isolation and PCR amplification of cpDNA *trnL* intron, *rps16-trnK* intergenic spacer and *atpB-rbcL* intergenic spacer regions

Genomic DNA was isolated from young leaves of each of the taxa using CTAB method^52^. The quality of DNA in each sample was checked by 1.0% (w/v) agarose gel electrophoresis. DNA concentration was measured using Eppendorf BioSpectrophotometer. The amplification of the cpDNA *trnL* intron region was performed in a programmable thermal cycler (Mastercycler Nexus, Eppendorf AG 22331 Hamburg) using the universal forward primer: 5′-CGA AAT CGG TAG ACG CTA CG -3′ and reverse primer: 5′-GGG GAT AGA GGG ACT TGA AC-3′, as described in Saha *et al*.^37^. PCR cycling conditions were followed according to Taberlet *et al*.^53^. For amplification of each of the cpDNA *rps16-trnK* intergenic spacer and *atpB-rbcL* intergenic spacer region, a 25 μl reaction was setup with 2.5 μl of 10X PCR buffer along with15 mM MgCl_2_ (Genei, Bangalore), 0.5 μl of 10 mM dNTP mix (Genei, Bangalore), ~100 ng template DNA, 0.5 μl of Taq DNA polymerase (5U/μl) (Genei, Bangalore) and 1 μl of each primer (4.0 pM/μl). Both the forward (F) and reverse (R) primers specific to cpDNA *rps16-trnK* intergenic spacer (F: 5′-AAA GTG GGT TTT TAT GAT CC-3′ and R: 5′-TTA AAA GCC GAG TAC TCT ACC-3′^35^) and *atpB-rbcL* intergenic spacer (F: 5′-ACA TCK ART ACK GGA CCA ATA A-3′ and R: 5′-AAC ACC AGC TTT RAA TCC AA-3′^54^) were commercially synthesized by GCC Biotech (India) Pvt. Ltd. Kolkata, India. The PCR cycling conditions were as follows: for *rps16-trnK* intergenic spacer: initial denaturation at 95 °C for 3 min followed by 30 cycles at 95 °C for 30 sec, annealing at 48 °C for 30 sec, extension at 72 °C for 1 min; final extension was at 72 °C for 8 min and for *atpB-rbcL* intergenic spacer: initial denaturation at 94 °C for 2 min followed by 30 cycles at 94 °C for 1 min, annealing at 50 °C for 1 min, extension at 72 °C for 1 min; final extension was at 72 °C for 8 min.

### PCR amplification of rDNA internal transcribed spacer region (ITS1-5.8S-ITS2)

For the amplification of rDNA ITS1-5.8S-ITS2 region, specific primers (forward primer: 5′-GAA TGG TCC GGT GAA GTG TTC GG-3′ and the reverse primer: 5′-CGC CTG ACC TGG GGT CGT G-3′) were designed using NCBI primer blast software (http://www.ncbi.nim.nih.gov) and were commercially synthesized by Integrated DNA Technologies (RFCL Limited, New Delhi, India). 25 μl PCR reaction mix contained 2.5 μl of 10X PCR buffer along with 15 mM MgCl_2_ (Genei, Bangalore), 1.0 μl of 10 mM dNTP mix (Genei, Bangalore), ~100 ng template DNA, 1.0 μl of Taq DNA polymerase (5 U/μl) (Genei, Bangalore) and 1 μl of each primer (4.0 pM/μl). The PCR cycling conditions were as follows: initial denaturation at 95 °C for 3 min followed by 30 cycles at 95 °C for 30 sec, annealing at 66 °C for 45 sec, extension at 72 °C for 1 min; final extension was at 72 °C for 8 min.

### DNA sequencing

All the PCR amplicons of cpDNA *trnL* intron, *rps16-trnK* intergenic spacer and *atpB-rbcL* intergenic spacer and rDNA ITS1-5.8S-ITS2 of the studied species of *Drimia* were sequenced using the Big Dye Terminator cycle sequencing method (Xcelris Labs Ltd, Gujarat, India; http://www.xcelrislabs.com). Chromatograms of all the DNA sequences were analyzed by using Bio-Edit.v.7.1.3 software (Ibis Biosciences, Carlsbad, CA 92008). Multiple sequence alignments were performed using ClustalW (http://www.genome.jp/tools/clustalw) with Gap Open Penalty: 15 and Gap Extension Penalty: 6.66. All the newly generated sequences have been deposited in the NCBI GenBank database (http://www.ncbi.nim.nih.gov) under accession numbers listed in Table 1.

### Phylogenetic analysis using cpDNA non-coding sequences

Phylogenetic analysis using cpDNA *trnL* intron sequences of 54 accessions representing six subfamilies of Asparagaceae and two outgroup taxa (Supplementary Table S2) was performed by maximum likelihood method using the partitioned model option with MEGA 6.06^55^. Based on Bayesian information criterion (BIC) and Akaike information criterion, corrected (AICc) using MEGA 6.06^55^, the best-fit nucleotide-substitution model was found to be T92 + G (Tamura 3-parameter model), with the lowest BIC score (5267.637), and lowest AICc score (4414.472). Initial tree(s) for the heuristic search were obtained automatically by applying Neighbor-Join and BioNJ algorithms to a matrix of pairwise distances estimated using the Maximum Composite Likelihood (MCL) approach, and then selecting the topology with superior log likelihood value (-2098.72). The tree was drawn to scale, with branch lengths measured in the number of substitutions per site. A discrete Gamma distribution was used to model evolutionary rate differences among sites [5 categories (+G, parameter = 1.1548)]. Positions containing gaps and missing data were eliminated from the datasets (complete deletion option). Bootstrapping of the datasets was performed with 1000 replications^56^.

In order to clarify the interspecies relationship among the studied members of *Drimia*, a phylogenetic analysis was further conducted using the concatenated sequences of cpDNA *rps16-trnK* intergenic spacer, *atpB-rbcL* intergenic spacer and cpDNA *trnL* intron following Farris *et al*.^57^. A total of 14 accessions including 2 outgroup taxa viz. *Asparagus officinalis* [Accession nos.: AB613992.1 (*rps16-trnK* intergenic spacer), AY147755.1 (*atpB-rbcL* intergenic spacer), KJ774036.1 (*trnL* intron)] and *Asparagus setaceus* cultivar Pyramidalis [Accession nos.: AB613995.1 (*rps16-trnK* intergenic spacer), JF784417.1 (*atpB-rbcL* intergenic spacer), KJ774038.1 (*trnL* intron)] were aligned with the ClustalW programme (with Gap Open Penalty: 15 and Gap Extension Penalty: 6.66) in the MEGA 6.06 package^55^. The phylogenetic analysis of the aligned matrix was performed by both maximum likelihood (ML) and maximum parsimony (MP) methods. The T92 model (Tamura 3-parameter model) of nucleotide-substitutions for the ML analysis of the concatenated sequences of three cpDNA non-coding sequence data was determined by the lowest BIC (8118.609) and AICc scores (7897.829). The Subtree-Pruning-Regrafting (SPR) algorithm^58^ with search level 1 was used to obtain the MP tree. The initial trees were obtained by the random addition of sequences (10 replicates). Bootstrap analyses were performed on 1000 replicates^56^.

### Phylogenetic analysis using rDNA ITS1-5.8S-ITS2 sequence

Phylogenetic analysis using the rDNA ITS1-5.8S-ITS2 sequence was also performed for further confirmation of the degree of relatedness among the studied members of *Drimia*. A total of 14 accessions including 2 outgroup taxa (*Asparagus officinalis* Accession no. KJ868767.1 and *Asparagus setaceus* cultivar Pyramidalis Accession no. KJ885623.1) were aligned with the clustalW programme with gap open penalty 15 and gap extension penalty 6.66 (http://www.genome.jp/tools/clustalw). The phylogenetic analysis was done by ML method with MEGA 6.06^55^ as mentioned in the above. The best-fit nucleotide-substitution model was found to be T92 + I (Tamura 3-parameter model), with the lowest BIC score (3793.582), and lowest AICc score (3601.744). The bootstrap method was employed with 1000 replications^56^.

### Morphological and anatomical characterization of leaf

A minimum of 10 mature leaves from three separate individual plants of each taxon of the genus *Drimia* was used for evaluating different qualitative and quantitative morphological and anatomical parameters (Supplementary Table S1). The morphological parameters included shape of leaf (LS), number of leaves per bulb (LN), leaf length (LL) and width (LW). LL was measured from base to tip of the fully expanded leaf blade while LW was measured from margin to margin at the middle portion of the leaf blade. For leaf anatomical studies, free-hand cross sections were prepared from the middle part of the leaf with a sharp razor blade. At least three sections were used for analysis and scoring of different anatomical characters viz. shape (LS_T.S_) and thickness (LT_T.S_) of leaf in t.s., type (PT) and length (LPC) of palisade cells according to Saha *et al*.^37^ and Chatterjee *et al*.^59^. Sections were observed under Leitz BioMed compound microscope and photographed with the attached ProgRes CT5 digital image documentation system. The observed qualitative characters (LS, LS_T.S_ and PT) were converted into character states (binary and multistate) and were finally scored for each taxon (Supplementary Table S1). The experiment was repeated thrice.

### Stomatal characterization

For stomatal characterization, a minimum of 10 mature leaves from three separate individuals of each taxon of *Drimia* was studied. For uniformity, only the middle portion of the mature leaf was considered and epidermises were peeled off to analyse different stomatal characters (Supplementary Table S1) following the method described in Saha *et al*.^37^. Both the upper and lower epidermal peels were observed and photographed under Leitz BioMed compound microscope equipped with digital camera ProgRes CT5. Length (SL in µm) and width (SW in µm) of stomata, stomatal index (SI), length (EL in µm) and width (EW in µm) of the surrounding epidermal cells were measured from at least 10 stomatal complexes selected randomly. The stomatal index (SI) was determined by calculating the average number of stomata and the number of epidermal cells per microscopic field (area: 205892.61 µm^2^) following the protocol of Reginato *et al*.^60^. All kinds of measurements were done using the software package ProgRes Capture Pro 2.8.8 (Jenoptik Optical System). The experiment was repeated thrice.

### Scanning electron microscopic (SEM) analysis of pollen grains

The exine surface architectures of the pollen grains of three separate individuals of five collected species and 10 accessions of *Drimia* (*D*. *indica* population-I, II, III and IV, *D*. *coromandeliana*, *D*. *polyantha* population-I and II, *D*. *govindappae* and *D*. *wightii* population-I and II) were studied by SEM analysis following the protocol of Talbot and White^61^. Pollen grains were mounted on the aluminium stubs using a double adhesive carbon tape and sputter coated with a 20–30 nm thick film of Au/Pd under S150 Sputter Coater. The sample containing stubs were examined at 15 Kv accelerating voltage and photographed under a SEM-EDX unit (SEM-Carl Zeiss Evo-40 EDX- Oxford Instrumentation) [GSI, Geological Survey of India, Kolkata].

### Statistical analysis

Descriptive statistics including means and standard errors and one-way analysis of variance (ANOVA) was carried out to test the significance of variation in the leaf traits of the studied taxon^62^. Tukey’s B multiple range tests was used for post hoc analyses. The statistical analysis was conducted at 0.05 probability level using SPSS v16.0 statistical package. To determine the interspecies relationship among the members of *Drimia* based on morphological, anatomical, and stomatal characters, cluster analysis was conducted on the Euclidean distance matrix with the unweighted pair group method using arithmetic averages (UPGMA) with default data transformation and normalization option with the InfoStat version 2013d (Free version) software package. To calculate the average Euclidean distance, a combination of the observed variables (morphological, anatomical and stomatal) per accession was analysed, which included: eight quantitative (LN, LPC, LT_T.S_, SL, SW, SI, EL and EW), two binary (LS_T.S_ and PT) and one multistate (LS) characters (Supplementary Table S1).

### Ancestral state reconstruction

To study the evolution of the leaf and pollen characters among the Indian species of *Drimia*, two important traits, viz. type of palisade cell (PT) and pollen exine pattern (PEP) were selected based on the earlier reports^19,30,39,41^. The character states of PT and PEP (Supplementary Table S1) of each taxon were used to reconstruct their ancestral states using Mesquite 3.31 software^63^. This software analyses the character state at the terminal taxa and graphically represents the history of character evolution. A phylogenetic tree was reconstructed based on combined sequence data from the three chloroplast non-coding DNA segments (cpDNA *trnL* intron, *rps16-trnK* intergenic spacer and *atpB-rbcL* intergenic spacer) using Mesquite heuristic search method. Alignment of the input sequences was done using Muscle 3.8.31 programme. The obtained phylogenetic tree was then served as a backbone to study the transition parameters for ancient and recent state reconstruction of morphological traits (PT and PEP) using maximum parsimony method.

## Supplementary information

Combined Supplementary Table S1 and S2

Combined Supplementary Figures S1, S2, S3 and S4

## Data Availability

All data generated or analysed during this study are included in this published article and its Supplementary Information files.
