# Supplementary material for: A molecular phylogeny of the genus Drimia (Asparagaceae: Scilloideae: Urgineeae) in India inferred from non-coding chloroplast and nuclear ribosomal DNA sequences
Source: Sci Rep. 2019 May 17;9:7563. doi: 10.1038/s41598-019-43968-z (PMC6525161; doi:10.1038/s41598-019-43968-z)
Supplement: Supplementary file 1 — Combined Supplementary Table S1 and S2 [file 41598_2019_43968_MOESM1_ESM.pdf]

A molecular phylogeny of the genus *Drimia* (Asparagaceae: Scilloideae: Urgineeae) in India inferred from non-coding chloroplast and nuclear ribosomal DNA sequences

Partha S. Saha, Sumita Jha*

Table S1 Thirteen morphological, anatomical and stomatal characters of leaves and one pollen morphological character and their abbreviations and states for the *Drimia* species

No.	Character	Abbreviation	Character state
	**Morphological:**		
1.	Shape of leaf	LS	Lanceolate-straight (0); Linear (1) and Lanceolate-curled (2)
2.	Number of leaves per bulb	LN	-
3.	Leaf length	LL	-
4.	Leaf width	LW	-
	**Anatomical:**		-
5.	Shape of leaf in t.s.	$LS_{T.S}$	Subulate: (0); Polygonal: (1)
6.	Type of palisade cells	PT	Columnar: (0); Spherical: (1)
7.	Length of palisade cells	LPC	-
8.	Thickness of leaf in t.s.	$LT_{T.S}$	-
	**Stomatal:**		
9.	Stomatal length	SL	-
10.	Stomatal width	SW	-
11.	Stomatal index	SI	-
12.	Epidermal cell length	EL	-
13.	Epidermal cell width	EW	-
	**Pollen morphology:**		
14.	Exine ornamentation pattern	PEP	Perforate: (0); Reticulate: (1)

Table S2 List of accessions used for cpDNA *trnL* intron-based phylogeny

Sl. No.	Species	GenBank Accession ID
Ingroup		
1.	*Agave attenuata*	DQ500898.1
2.	*Androstephium coeruleum*	AJ311071.1
3.	*Arthropodium milleflorum*	AJ232436.1
4.	*Asparagus aethiopicus*	KJ774031.1
5.	*Asparagus africanus*	KJ774039.1
6.	*Asparagus densiflorus*	KJ774034.1
7.	*Asparagus falcatus*	KJ774030.1
8.	*Asparagus gonoclados*	KJ774033.1
9.	*Asparagus setaceus* cultivar Pyramidalis	KJ774038.1
10.	*Asparagus racemosus*	KJ774032.1
11.	*Asparagus retrofractus*	KJ774035.1
12.	*Asparagus setaceus* cultivar Robustus	KJ774037.1
13.	*Beschorneria calcicola*	DQ500911.1
14.	*Brodiaea jolonensis*	AF117017.1
15.	*Camassia cusickii*	AJ232450.1
16.	*Charybdis maritima*	AJ232466.1
17.	*Chlorogalum palmatum*	AJ232448.1
18.	*Dasylirion acrotrichum*	AJ441179.1
19.	*Dichelostemma multiflorum*	AJ311078.1
20.	*Dracaena cochinchinensis*	JN377588.1
21.	*Drimia coromandeliana*	See Table 1 (Present study)
22.	*Drimia govindappae*	
23.	*Drimia indica* population-I	
24.	*Drimia indica* population -II	
25.	*Drimia indica* population -III	
26.	*Drimia indica* population -IV	
27.	*Drimia nararjunae*	
28.	*Drimia polyantha* population -I	
29.	*Drimia polyantha* population -II	
30.	*Drimia razii*	
31.	*Drimia wightii* population -I	
32.	*Drimia wightii* population -II	
33.	*Drimia maritima*	AJ247506.1

34.	*Drimia sanguinea*	AJ247510.1
35.	*Drimia undata*	AJ247512.1
36.	*Eustrephus latifolius*	KC428513.1
37.	*Furcraea macdougallii*	DQ500908.1
38.	*Hesperoyucca whipplei*	DQ500912.1
39.	*Liriope spicata*	KF671380.1
40.	*Maianthemum henryi*	EU850176.1
41.	*Manfreda potosina*	DQ500901.1
42.	*Milla biflora*	AJ311081.1
43.	*Muilla maritima*	AF117019.1
44.	*Nolina microcarpa*	AJ441178.1
45.	*Ophiopogon tonkinensis*	KF671373.1
46.	*Polianthes densiflora*	DQ500905.1
47.	*Polygonatum cyrtonema*	EU850170.1
48.	*Reineckea carnea*	AJ441182.1
49.	*Sansevieria metallica*	AJ441171.1
50.	*Thysanotus virgatus*	AJ232437.1
51.	*Triteleia lemmonae*	AJ311085.1
52.	*Yucca rigida*	DQ500916.1
Outgroup		
53.	*Tradescantia pallida*	AM113705.1
54.	*Weldenia candida*	AJ387746.1
