# Supplementary material for: A molecular phylogeny of the genus Drimia (Asparagaceae: Scilloideae: Urgineeae) in India inferred from non-coding chloroplast and nuclear ribosomal DNA sequences
Source: Sci Rep. 2019 May 17;9:7563. doi: 10.1038/s41598-019-43968-z (PMC6525161; doi:10.1038/s41598-019-43968-z)
Supplement: Supplementary file 2 — Combined Supplementary Figures S1, S2, S3 and S4 [file 41598_2019_43968_MOESM2_ESM.pdf]

A molecular phylogeny of the genus *Drimia* (Asparagaceae: Scilloideae: Urgineeae) in India inferred from non-coding chloroplast and nuclear ribosomal DNA sequences

Partha S. Saha, Sumita Jha*

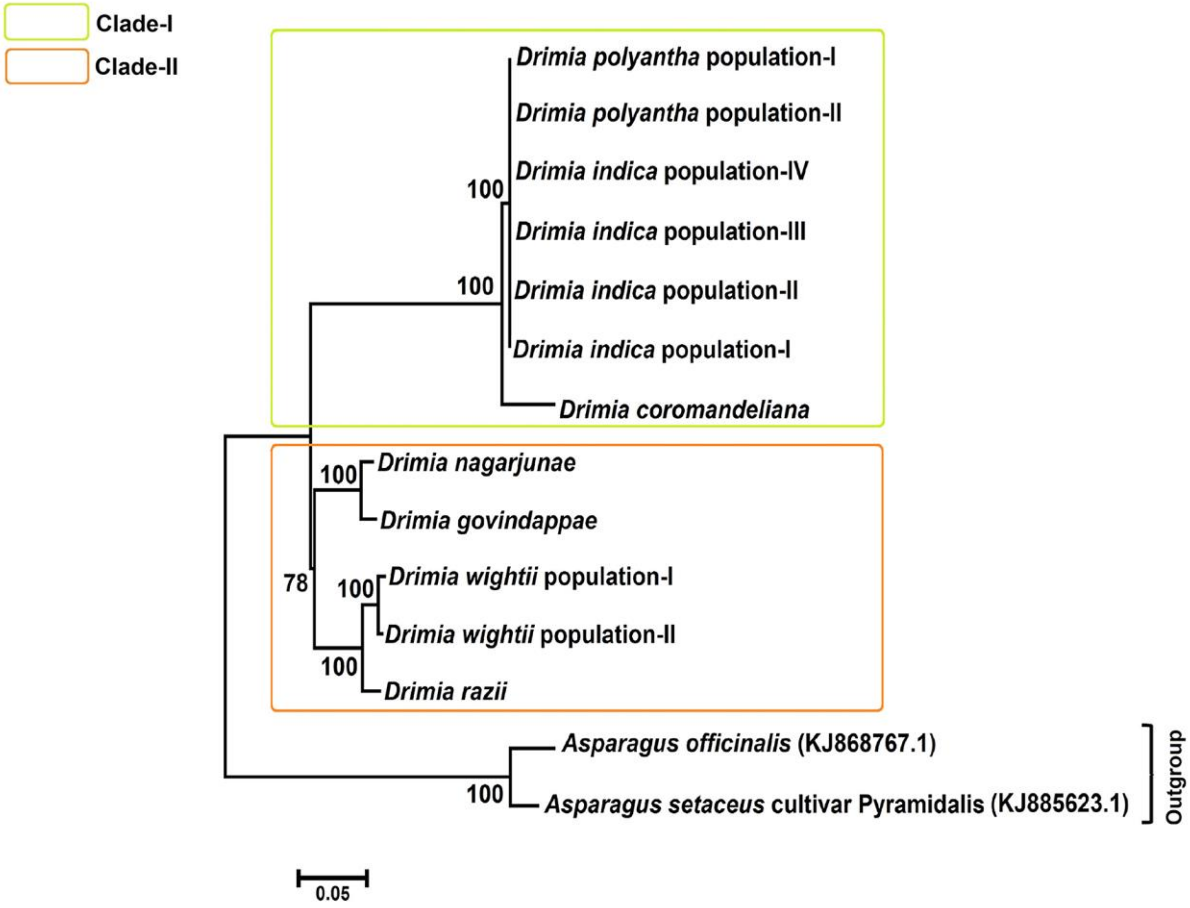

Fig. S1 Maximum likelihood tree showing the phylogenetic relationships among the Indian species of *Drimia* based on rDNA ITS1-5.8S-ITS2 sequences.

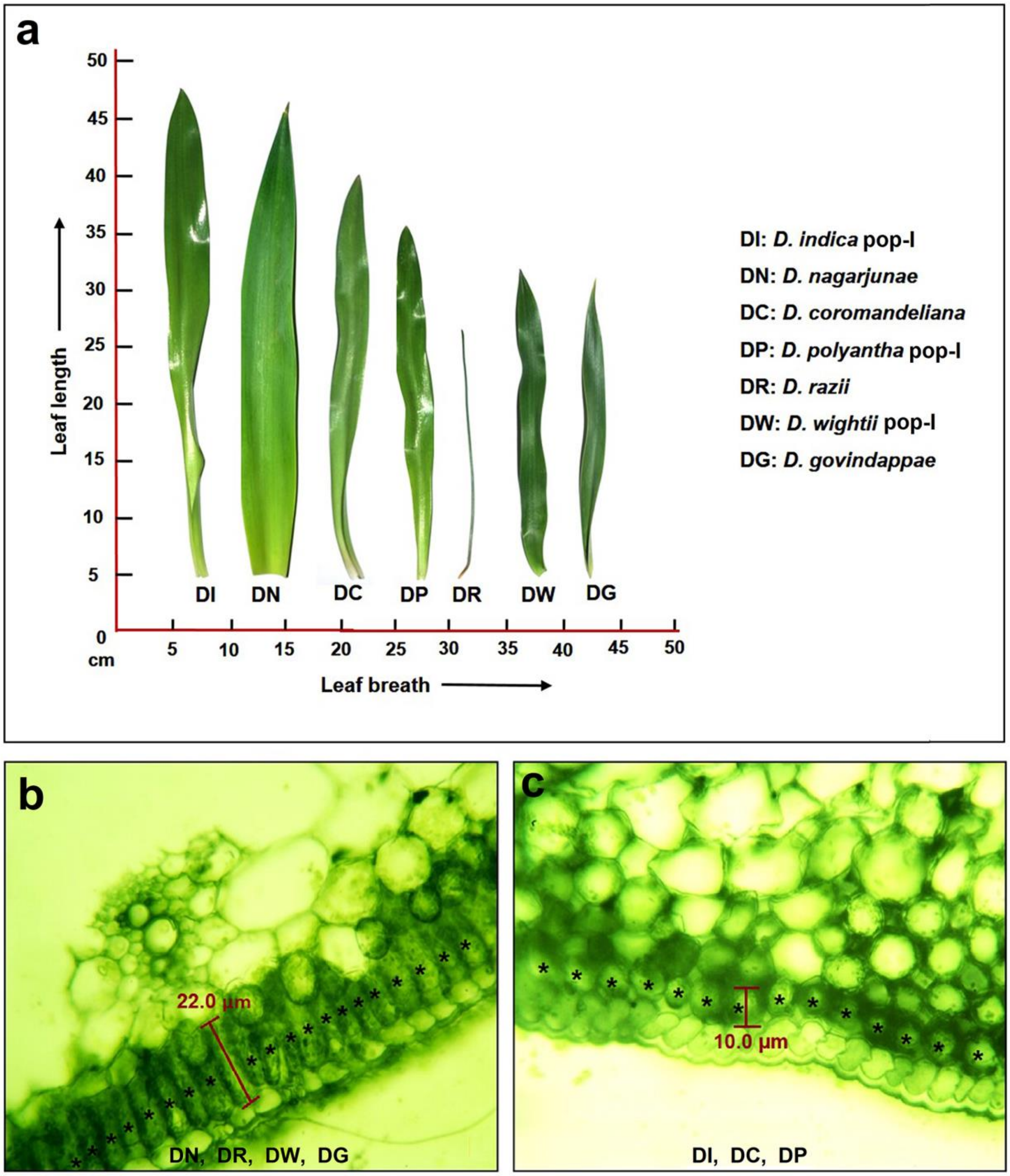

Fig. S2 a) Diversity in the leaf shapes among seven Indian species of *Drimia*, b and c) Representative photos of two types of palisade cells (columnar and spherical respectively) marked by stars (*) in the transverse section of *Drimia* leaves.

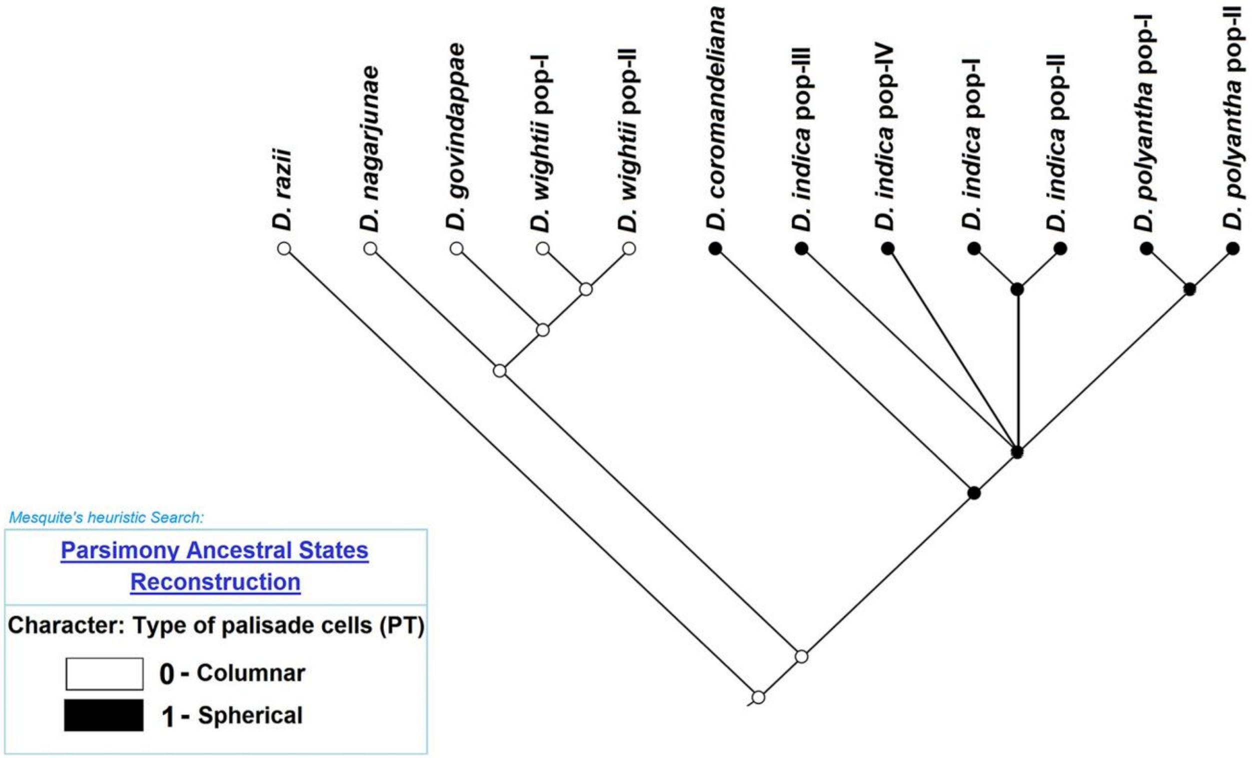

Fig. S3 Inference of evolution of type of palisade cells for Indian species of *Drimia* based on cpDNA concatenated non-coding sequence based phylogenetic tree.

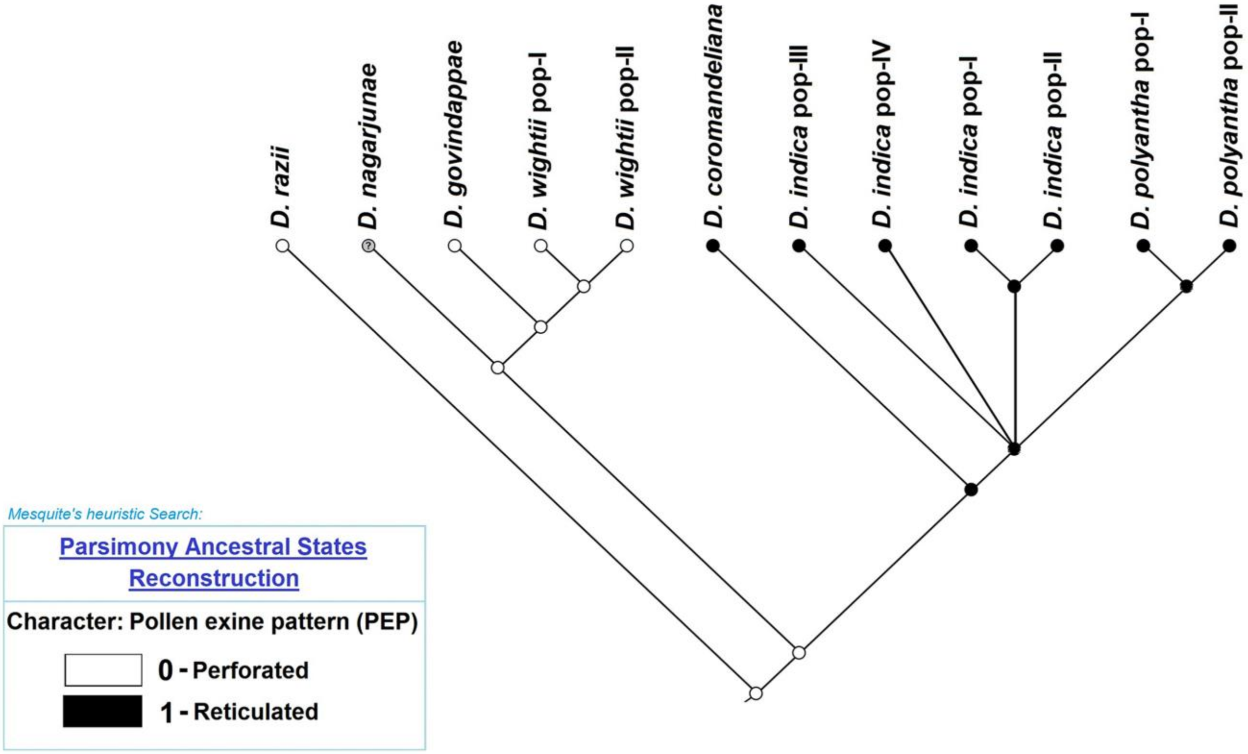

Fig. S4 Inference of evolution of pollen exine ornamentation pattern for Indian species of *Drimia* based on cpDNA concatenated non-coding sequence based phylogenetic tree
